# Effect of extremely low-frequency magnetic fields on light-induced electric reactions in wheat

**DOI:** 10.1080/15592324.2021.2021664

**Published:** 2022-01-07

**Authors:** Marina Grinberg, Maxim Mudrilov, Elizaveta Kozlova, Vladimir Sukhov, Fedor Sarafanov, Andrey Evtushenko, Nikolay Ilin, Vladimir Vodeneev, Colin Price, Evgeny Mareev

**Affiliations:** a Department of Biophysics, Lobachevsky State University of Nizhny Novgorod Lobachevsky State University of Nizhny Novgorod, Nizhny Novgorod, Russia; b Department of Geophysical Electrodynamics, Institute of Applied Physics, Russian Academy of Sciences, Nizhny Novgorod, RussiaRussia; c Porter School of the Environment and Earth Sciences, Tel Aviv University, Tel Aviv-Yafo, Israel

**Keywords:** extremely low frequency magnetic ﬁeld, Schumann resonance, light-induced electric reactions, calcium, wheat

## Abstract

Magnetic field oscillations resulting from atmospheric events could have an effect on growth and development of plants and on the responsive reactions of plants to other environmental factors. In the current work, extremely low-frequency magnetic field (14.3 Hz) was shown to modulate light-induced electric reactions of wheat (*Triticum aestivum* L.). Blue light-induced electric reaction in wheat leaf comprises depolarization and two waves of hyperpolarization resulting in an increase of the potential to a higher level compared to the dark one. Fluorescent and inhibitory analysis demonstrate a key role of calcium ions and calcium-dependent H^+^-ATPase of the plasma membrane in the development of the reaction. Activation of H^+^-ATPase by the increased calcium influx is suggested as a mechanism of the influence of magnetic field on light-induced electric reaction.

## 1. Introduction

All the living organisms of the Earth live and develop in the magnetic field that is not uniform. There are areas of elevated magnetic field (MF) strength in the frequency spectrum, so-called Schumann resonance frequencies. Their maxima are located at 7.8, 14.3, 20.8, 27.3, and 33.8 Hz. The source of these fields with Schumann resonance frequencies is global lightning activity, and the electromagnetic waves emitted from lightning channels with some vertical component of charge transfer.^1,2^ Owing to very large wavelengths, they have essentially global nature, and even in fair-weather regions, living organisms are permanently surrounded by electromagnetic oscillations at Schumann resonance frequencies. Extremely low-frequency MF (ELFMF) strength on the whole and, particularly, at Schumann resonance frequencies, can change in a broad range via transient processes in the ionosphere.^3,4^ Such oscillations themselves and in combination with other fluctuating environmental factors (significant changes of illumination, humidity, etc.) can affect the functioning of living organisms.

It is well known that magnetic fields, including ELFMF, affect growth and development of animals and plants. Numerous studies demonstrate that magnetic fields have an effect on such integral parameters of plant growth and development as germination rate, size, biomass, productivity.^5–8^ The basis of these effects has been partly studied at the level of physiological processes. Thus, for one of the most important physiological processes for plant functioning, photosynthesis, the effect of low- and extremely low-frequency MF of varying intensity on a number of parameters, including assimilation of СО_2_, photosynthetic pigment content, activity of light stage reactions, was shown.^9–12^ Deciphering the mechanisms determining the biological effects of MF on plants are impeded by a high degree of interaction and mutual effects of physiological processes, as well as by the low level of knowledge about a number of processes that could potentially be sensitive to (electro)magnetic field. One of such processes is, for example, the generation of electric potential.

Electric potential of cell membrane performs a number of essential functions. The most important of them are regulation and energization transmembrane substance transport and informational and signaling function resulting in triggering intra- and intercellular signaling cascades. Change of electric potential of the membrane accompanies and mediates the effects of numerous environmental stimuli, such as changes of illumination, humidity, temperature, etc.^13–16^

Light-induced shifts of electric potential comprise transient depolarization that could induce action potential (AP) followed by hyperpolarization. Such changes of electric potential are related to alteration of transmembrane ion flow caused by changed activity of ion channels and pumps.^13,16–20^ Light-induced electric reactions were studied for many plant species of different taxonomic groups, from algae and mosses to flowering plants, including Arabidopsis, beans, pea, tobacco, cucumber, corn, etc.^13,18,19,21–25^ General properties of the formation and the spectral dependence of the light-induced reaction were revealed, although species-specific features of the responses may also take place. Particularly, it was shown that AP arising after illumination could be induced by red, white and blue light.^17,18,20^ However, as a rule, only blue and white light causes the formation of further multiphase responses, leading to stable hyperpolarization with preceding transient depolarization in response to light switching on and stable depolarization with preceding transient hyperpolarization in response to light switching off.^20^ Ca^2+^, H^+^, Cl^−^ and К^+^ are considered to be the key ions responsible for progression of light-induced reactions.^19,24–27^

The effects of MF on ion transport and electric potential generation in animal cells revealed earlier^28,29^ point onto the potential possibility of effect of MF onto electrogenesis in plants. Certain works explain experimentally observed effects of MF, such as effect on leaf motion in locomotor plants, right by action onto electrogenesis.^30^ A recent study showed that distribution of electric signals in plants produces magnetic fields.^31^ This shows the possibility of the reverse effect, such as the influence of external MF on electric reactions in plants. However, the effects of ELF magnetic field on electric reactions of plants induced by natural fluctuations of environmental factors have not been studied yet. The object of the present study is the effect of ELFMF with a Schumann resonance frequency on light-induced electric reaction and mechanisms of such an effect.

## 2. Materials and methods

### 2.1. Growth and exposure of plants in the magnetic field

The experiments were carried out on 14–16-days-old wheat seedlings (*Triticum aestivum* L.), variety «Daria». The plants were grown in soil-containing vessels at 24°C with a 16-h light/ 8-h dark cycle. Some experiments were performed on detached second leaves 15 cm long.

**Figure 1.**
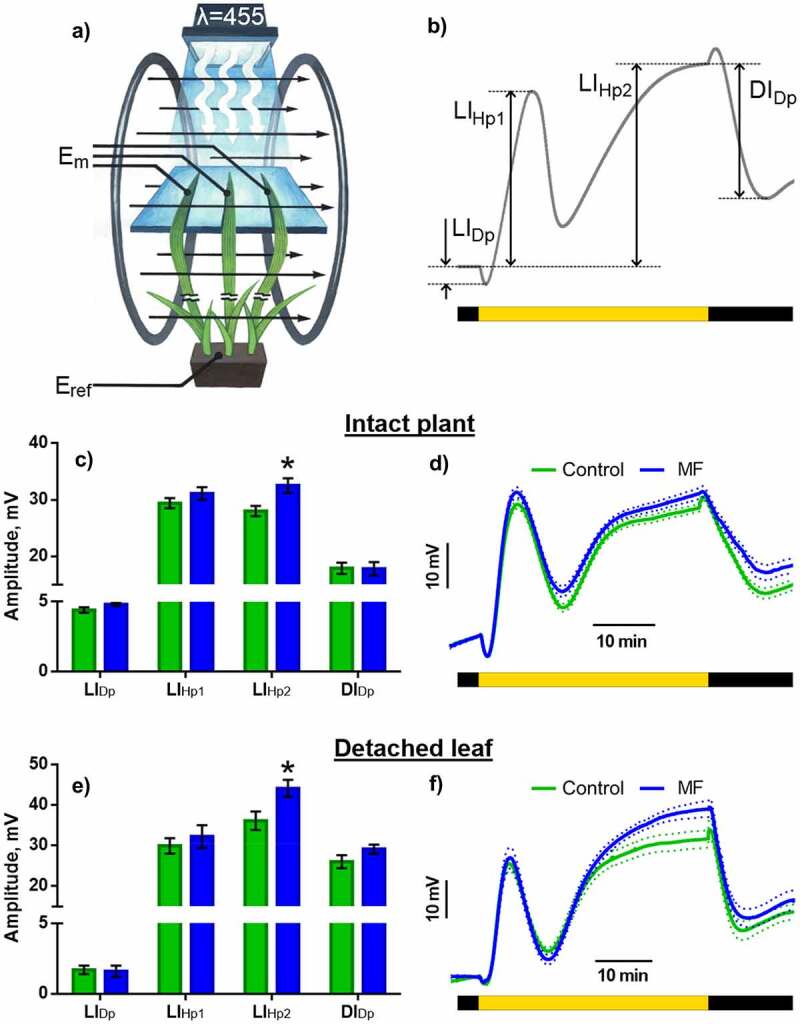
Effect of extremely low-frequency MF on the parameters of light-induced electric reaction of the wheat seedlings. The control is green, the MF is blue. a – Scheme of the experimental setup. E_m_, measuring electrodes; E_ref_, reference electrode. b – Light-induced electric reaction with key stages denoted. LI_Dp_, light-induced depolarization; LI_Hp1_, the first light-induced hyperpolarization; LI_Hp2_, the second light-induced hyperpolarization; DI_Dp_, dark-induced depolarization; c – amplitudes of the key phases and d – averaged curves of light-induced reactions in control and under MF action in whole plants; e – amplitudes of key phases and f – Averaged curves of light-induced reactions in control and under MF action in detached leaves. * – р < 0.05.

System for plant treatment by artificial ELFMF was assembled according to previous research;^11^ it is based on Helmholtz coils (100 loops per each coil with corrected input impedance of 50 Ohm) with 0.3 m radii (volume of homogenous magnetic field was about 20 × 20 × 20 cm^3^) (Figure 1). RIGO DG1032 Waveform Generator (RIGOL Technology Co., Ltd., Suzhou, China) was used for the generation of sinusoidal electrical signals with frequency equal to 14.3 Hz. Magnitude of MF was 9 µT. The parameters of MF acting on the plants were chosen based on the most pronounced effects found during the analysis of frequency-amplitude dependency of the effect ELFMFs on photosynthesis, performed earlier.^11,12^

### 2.2. Methods

#### 2.2.1. Registration of light-induced electric reactions

Surface potentials were measured by means of a set of Ag^+^/AgCl macroelectrodes arranged on the second leaf of a whole plant and on a detached second leaves 15 cm long. The reference electrode was located in wet soil (in the case of whole plants) or in the vessel in which the cut margin of the detached leaf was immersed. The electrodes contacted the leaf through a thread wetted with a standard solution (0.5 mM NaCl, 1 mM KCl, 0.5 mM CaCl_2_). The data were recorded by the high-impedance amplifiers IPL-113 (Semico, Russia) and then processed on a PC in Param2 program.

The membrane potential was recorded in the second leaf at a distance of 5–7 cm from the tip in mesophyll cells. The measurements were taken using a SliceScope Pro 2000 electrophysiological appliance (Scientifica, UK). Micropipettes with a resistance of 12–15 MΩ and a tip of about 1 μm diameter were manufactured on a P-97 puller (Sutter Instrument, USA). The micropipette was inserted into the cell using motorized PatchStar manipulators under visual control. The reference electrode was placed in a standard solution washing the studied area of the leaf. The data were processed by a Multiclamp 700B amplifier and Digidata1550 data acquisition system.

Changes in the electric membrane potential were recorded in response to the on-off light in plants in the presence and in the absence of MF. Dark adaptation before recording the light-induced reaction was 40 min. The period of illumination was 40 min. After switching off the light, registration of electrical potential continued for 20 min. Plant leaves were illuminated by LED matrix with 455 nm wavelength, with an intensity of 625 µmol m^−2^s^−1^. A part of leaf with measuring electrode was illuminated. Reference electrode contacted with the root in whole plant experiments or with the unilluminated leaf base in detached leaf experiments (Figure 1). Magnetic field was turned on at the 20th minute of dark adaptation, and it was acting during the whole recording period. Control plants were subjected to similar procedures in the absence of MF.

#### 2.2.2. Inhibitory analysis

The following agents were used in inhibitory analysis: EDTA, 2 mM; CaCl_2_, 5 mM; Na_3_VO_4_, 4 mM; NaN_3_, 1 mM; TEA, 1 mM. The inhibitors were loaded onto the 15-cm long second leaves detached from the plant. The cut margin of the leaf was immersed into a vessel with inhibitor, after that the leaf was incubated under decreased pressure (0.2 atm) for 5 min. The detached leaves of the control plants were immersed into standard solution and underwent all the same procedures. The period between inhibitor loading and light-induced reaction recording was 60 min.

#### 2.2.3. Registration of Са^2+^dynamics

Dynamics of Са^2+^ during light-induced reaction was registered in the detached second leaves using Fluo-4 AM fluorescent probe. To increase the effectiveness, probe loading was performed at low temperature during three cycles of incubation at decreased air pressure (0.2 atm for 5 min with 5 min intervals between the cycles). The detached leaves of the control plants were immersed into standard solution and underwent all the same procedures.

Fluorescence registration was recorded on the whole leaves using fluorescence imaging system DVS-03 (ILIT RAS, Russia), with maximal image acquisition area 15 × 15 cm. Adaptation period before the measurements was 60 min. Illumination parameters were similar to those used in registration of electrophysiological reactions. Dark adaptation before recording the light-induced reaction was 40 min. Illumination period was to 40 min. Fluo-4 AM fluorescence was recorded using 530/50 interference filter (Zeiss AG, Germany). The images were recorded every 5 s, exposure was 40 ms. During the analysis of fluorescent images on the recorded leaf, regions of interest were selected, and fluorescence intensity was determined as the difference between the signals of plants with and without the probe. Electric activity was registered in parallel with fluorescence registration.

#### 2.2.4. Statistics

Each series of experiments consisted of 6–22 repetitions; every replicate was performed on a separate plant. The mean and standard error of mean were calculated, and the normal data distribution was confirmed for all the experiments; the significance of differences was evaluated by Student t-test. Typical and averaged records are also presented in the results.

## 3. Results

### 3.1. Effect of magnetic field on light-induced electric reaction

Formation of light-induced electrical reaction of wheat plants occurs in several stages (Figure 1). The short depolarization stage (Light-Induced DePolarization, LI_Dp_) begins right after the start of illumination, and it has an amplitude of several mV. Some plants demonstrated AP-like reaction with an amplitude of several dozens of mV. The first wave of light-induced hyperpolarization (LI_Hp1_) is developing after depolarization for around 10 min (Figure 1). Hyperpolarization amplitude reaches several dozens of mV in its maximum, after that the potential decreases, becoming closer to the dark level. The second wave of light-induced hyperpolarization (LI_Hp2_) is slower, and the new stationary level of potential is reached in several dozens of minutes. The reaction on switching off the light has also several stages. A short-term (2–3 min) hyperpolarization with several mV amplitude takes place right after the end of illumination. This is followed by dark-induced depolarization (DI_Dp_) and the potential slowly reaches the dark level.

**Figure 2.**
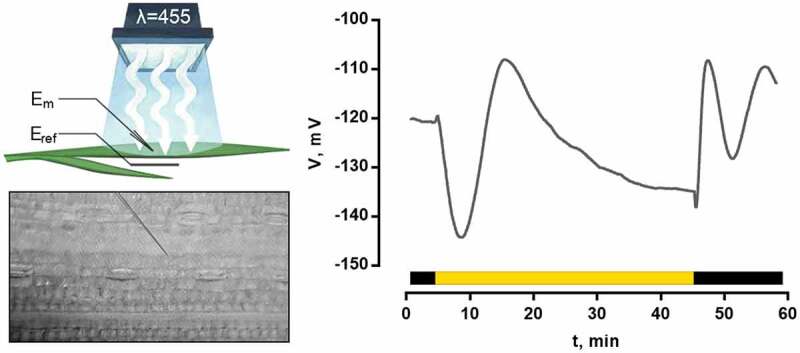
A typical blue light-induced reaction registered by microelectrode recording system from a wheat leaf mesophyll cell. The measurement scheme is presented on the left. E_m_ – measuring electrodes; E_ref_ – reference electrode.

For the purpose of examination of correspondence between reactions recorded by macroelectrodes from leaf surface and changes of cell membrane potential, the light-induced electrical reactions were registered by microelectrode recording system. It was shown (Figure 2) that light-induced changes of membrane potential of wheat mesophyll cells include all the same stages as changes of surface electric potential, and there is a good agreement between the records (Figures 1 and 2).

Action of ELF magnetic field does not change stationary potential level and the character of progression of light-induced electric reaction, but it affects amplitudes of certain stages (Figure 1). The most significantly affected stage is long-term hyperpolarization (LI_Hp2_) determining the stationary level of potential in the light. Hyperpolarization amplitude in control plants comprises 28.0 ± 0.9 mV, whereas under the action of MF it increases up to 32.5 ± 1.3. Upon development of dark-induced electric reaction in plants, hyperpolarization peak almost disappears under MF. Meanwhile, the amplitude of dark-induced depolarization is not affected significantly.

For further analysis of the mechanisms of revealed ELFMF effects, the experiments were carried out in model system of detached leaves loaded with fluorescent probe or inhibitors. To examine the relevance of such a model, experiments with light-induced reaction registration were also carried out on leaves loaded with standard solution. The character and tendencies of light-induced reactions specific for the whole plant are also retained in the detached leaves (Figure 1). The key effects of magnetic field, including enhancement of the second light-induced hyperpolarization wave (LI_Hp2_) and disappearance of hyperpolarization peak in the dark, are also absolutely reproducible for this model system.

### 3.2. Light-induced [Са^2+^]_cyt_ dynamics

**Figure 3.**
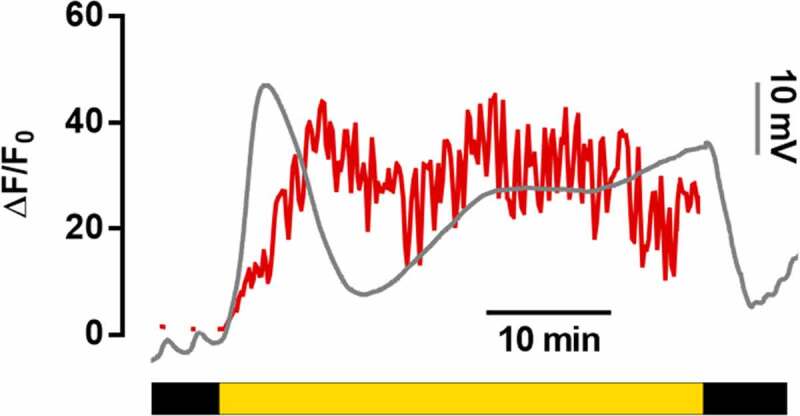
A typical record of [Ca^2+^]_cyt_ concentration dynamics accompanying blue light-induced electric reaction. Red line, dynamics of ΔF/F_0_; gray line, dynamics of electric potential recorded from the same leaf.

To determine the dynamics of intracellular calcium concentration during light-induced electric reaction, fluorescence of Fluo-4 probe was recorded. The increase of fluorescence intensity of the probe corresponds to elevation of [Ca^2+^]_cyt._ (Figure 3).

Dynamics of [Ca^2+^]_cyt_ has a biphasic nature. Right after switching on the light, rapid accumulation of calcium in cytoplasm begins, coinciding in time with the development of the first hyperpolarization wave (LI_Hp1_) (Figure 3). The second [Ca^2+^]_cyt_ wave has a smaller amplitude and prolonged duration. The wave development time corresponds to the second hyperpolarization wave (LI_Hp2_), but, in contrast to electric potential, calcium wave appears to be reversible, and it is attenuated with time. Fluorescent analysis used in the work does not allow to estimate dark-induced [Ca^2+^]_cyt_, dynamics, because the method requires periodic illumination of the plant.

### 3.3. Effect of inhibitors on light-induced electric reactions

To reveal the mechanisms of action of ELFMF on light-induced electric reactions, inhibitory analysis was performed. Inhibitors affecting transport of Ca^2+^, K^+^ and H^+^ ions were used.

**Figure 4.**
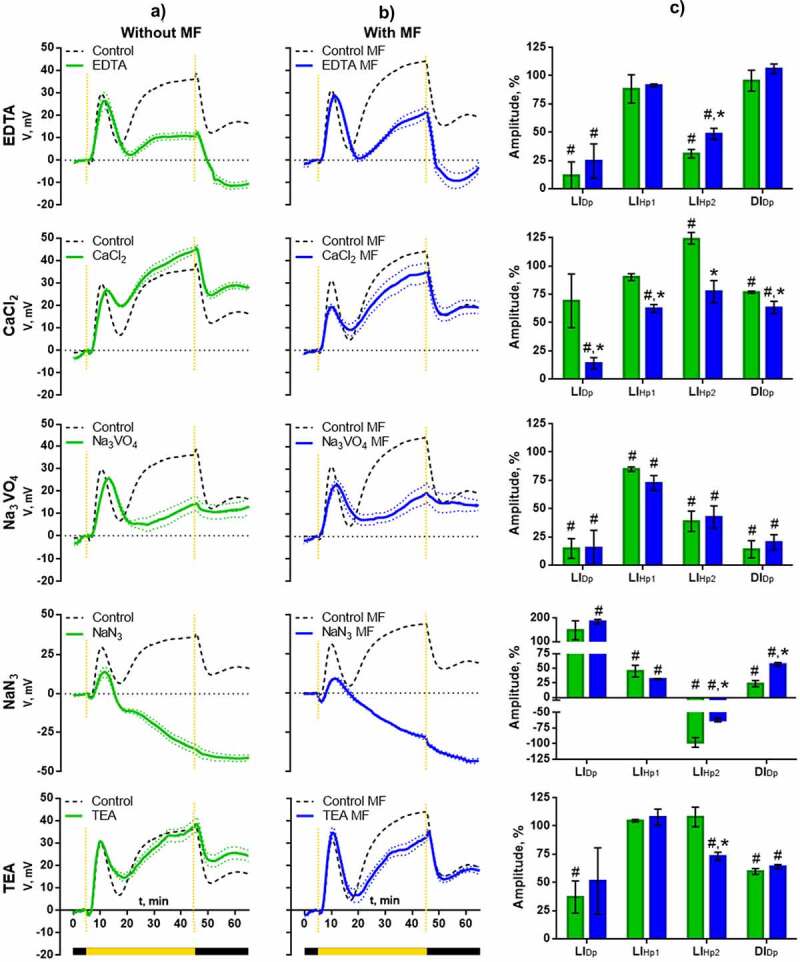
Effect of extremely low-frequency MF on the parameters of light-induced electric reaction of wheat seedlings in presence of inhibitors. The control is green, the MF is blue. a – Averaged records of the reactions in absence of the field compared to control without inhibitors. b – Averaged records of the reactions under MF compared to control without inhibitors. c – Diagrams of amplitude of the key phases of light-induced reactions as percentage of control amplitude. The dotted lines indicate the on and off of the light. # – Significant difference from inhibitor-free control, p < 0,05. * – Significant difference between groups in absence and in presence of MF, р < 0,05.

Calcium ion chelator, EDTA, partially suppresses the progression of light-induced depolarization (LI_Dp_) and significantly attenuates the second hyperpolarization wave (LI_Hp2_): amplitude of LI_Hp2_ in presence of EDTA comprises 31 ± 3.7% of control values. Also, deceleration of the development of this stage of light-induced electric reaction takes place. Turning off the lights induces the formation of deeper depolarization in presence of EDTA compared to control. MF partially compensates the effect of EDTA on light-induced electric reaction: suppression of LI_Dp_ amplitude is less prominent, suppression of LI_Hp2_ amplitude is significantly lower (residual amplitude comprises 31 ± 3.7% without MF and 48.6 ± 4.9% under MF), the depth of dark-induced depolarization is decreased. (Figure 4)

Elevated extracellular calcium concentration (5 mM instead of 0.5 mM CaCl_2_) has an effect opposite to EDTA: amplitude of LI_Hp2_ increases significantly and comprises 123.9 ± 4.5%, the time of development of this stage is reduced, depolarization in the dark becomes less deep. In presence of MF, the elevated calcium concentration suppresses all stages of the light-induced electric reaction (Figure 4).

To estimate the role of H^+^-ATPase of plasma membranes in formation of light-induced electric reaction, a specific inhibitor, Na_3_VO_4_, and the metabolic inhibitor reducing ATP content in the cell, NaN_3_, were used. These inhibitors most significantly affect the second wave of hyperpolarization: its progression is essentially suppressed in presence of Na_3_VO_4_ (by 61.3%) or blocked completely in presence of NaN_3_. Besides, NaN_3_ causes decrease of amplitude of the first peak of light-induced hyperpolarization by 54.9%. MF partially compensates the effect of H^+^-ATPase inhibitors on LI_Hp2_ stage (Figure 4).

The role of potassium was estimated using K^+^-channel inhibitor, TEA. The greatest effect of TEA acted on the processes occurring after switching off the light: the residual amplitude of DI_Dp_ comprises 59.7 ± 2.5%. In presence of MF, there are no differences in the effect of TEA on dark-induced reaction, but difference in amplitude of LI_Hp2_, which is decreased, exists (Figure 4).

## 4. Discussion

Despite the presence of numerous experimental studies in the literature, nowadays certain common knowledge on the ionic nature of light-induced electric reaction in plants is absent. On the whole, light-induced reaction can be described as transient depolarization capable of inducing AP with further plateau at the hyperpolarized level.^13,16,20^ Contribution of Ca^2+^, H^+^, Cl^−^ and К^+^ into generation of light-induced reaction was shown, but their role in formation of certain phases is not always clear; it apparently depends on both taxonomic group and species inside the group.^19,24,25^ The main contributor to depolarization is calcium, which induces activation of anionic channels and Cl^−^ efflux (depolarization). K^+^ effluxed after that restores the potential (repolarization). Prolonged hyperpolarization is formed mainly via activation of H^+^-ATPase, which is controlled by intracellular calcium.^13,19,24^ Light was shown to cause rapid elevation of calcium, which could take place as a two-wave process.^19,27^ High degree of synchronicity between Ca^2+^ and H^+^ fluxes was detected.^19^ Also, absence of contribution of potassium into the progression of light-induced hyperpolarization and its significant role in dark-induced response were shown.^20,26,27^

Our experiments demonstrated that light-induced electric reaction of wheat comprises depolarization and two hyperpolarization waves with elevation of potential to a higher value compared to the dark level (Figures 1 and 2). According to the results of inhibitory analysis, influx of calcium into the cell is required for depolarization (Figure 4). Elevation of its concentration in the cytoplasm was recorded immediately after turning on the light (Figure 3), which is in accordance with the measurements of calcium flow in beans, corn and Arabidopsis.^24,25,27^ Blue light receptors, phototropins, are directly responsible for the induction of rapid calcium influx after the beginning of illumination.^13,32^ Depolarization in wheat has a small amplitude, which is its difference from other plants, where deep depolarization inducing AP is a typical component of the light-induced electric reaction.^17–20^

Hyperpolarization changing depolarization could potentially be mediated by active pumping of protons from the cytoplasm by H^+^-ATPase, efflux of potassium in case of its bias from the equilibrium potential and active pumping of anions into the cell.^19^ The analysis shows that the first wave of hyperpolarization in wheat plants is almost insensitive to changes of external calcium concentration (EDTA and 5 мМ CaCl_2_), inhibitors of potassium channels (TEA) and specific inhibitor of H^+^-ATPase (Na_3_VO_4_), but its amplitude is slightly decreased by sodium azide, which decreases the activity of all ATP-dependent transporters. It was earlier suggested^19^ that fast light-induced hyperpolarization is determined by Cl^−^ influx caused by the action of ionic pump.

The key contribution to formation of the second, prolonged hyperpolarization wave in wheat is made by activation of H^+^-ATPase, which is supported by the results of inhibitory analysis with sodium azide and sodium orthovanadate (Figure 4). Key role of protons in hyperpolarization was also demonstrated in a number of papers.^13,26^ There are different hypotheses on the mechanisms of light-induced activation of proton ATPase. Particularly, role of calcium-dependent phosphorylation of the enzyme in activation of proton pump in the light was demonstrated.^14,33^ This is confirmed in our experiments by decreased hyperpolarization amplitude in the presence of calcium chelating agent EDTA, as well as by elevation of the amplitude with increased calcium concentration. The dynamics of cytoplasmic calcium demonstrates two partially overlapping waves, the second of which coincides in time with the progression of H^+^-ATPase-dependent hyperpolarization (Figure 3). Another mechanism of light-induced activation is photosynthetic sugar-mediated phosphorylation. This process does not depend on calcium and specific light receptors such as phytochromes, cryptochromes and phototropins.^34^

Termination of illumination causes the reactions that appear to be opposite to light-induced reactions in their shape, and they seem to have different mechanisms of formation. According to the works,^20,24^ Ca^2+^ significantly contributes to dark-induced reactions, which is in good agreement with our results. At the deficiency of calcium, deeper depolarization takes place, whereas excessive calcium has the opposite effect (Figure 4). Also, Н^+^ and К^+^ make a notable contribution, and the blockade of the flux of these ions significantly decreases depolarization amplitude. A conclusion on more important role of potassium in dark reactions rather than in light-induced ones matches the results described in the literature.^20,26,27^

Extremely low-frequency MF has a stimulating effect on light-induced electric reactions in wheat. The effect comprises increase of the amplitude of prolonged hyperpolarization in the light (Figure 1), which is mainly related to activation of H^+^-ATPase. As mentioned above, H^+^-ATPase activity can be regulated during light-induced reactions in two manners: via phosphorylation induced by calcium or by photosynthetic sugars.

**Figure 5.**
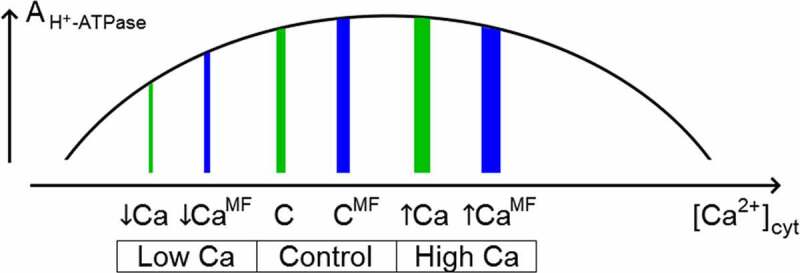
The suggested dependence of H^+^-ATPase activity on intracellular calcium concentration and levels of intracellular calcium in different conditions. C, control Ca^2+^ concentration (0.5 mM) in the solution; ↓Ca, decreased Ca^2+^ concentration provided by calcium chelator EDTA; ↑Ca, elevated Ca^2+^ concentration provided by excessive calcium content (5 mM) in the solution.

Our results may point at the increased calcium influx into cytosol under the action of MF. Decrease of the effect of calcium deficiency in ELF magnetic field supports this proposal (Figure 4). At elevated calcium concentration (5 mM СaCl_2_) ELFMF has an opposite effect. The observed effects determine the dome-like dependence of the activity of H^+^-ATPase on calcium concentration (Figure 5), because absence (EDTA) and excess (5 mM СaCl_2_) of calcium have similar influence. Similar trends were described earlier.^20^

Increase of calcium concentration is a well-documented effect of MF, including low-frequency MF, on plants and animals.^2,35,36^ This effect could be explained on the basis of the conventional view on the mechanisms of magnetic field reception. The most well-studied sensors perceiving magnetic field directly are blue light receptors, cryptochromes.^5,37,38^ Cryptochromes of higher plants are known to be able to participate in light-induced electrical reactions via activation of anionic channels,^32^ but, in contrast to phototropins, other blue light receptors, they are unable to cause calcium influx or directly affect proton ATPase.^39,40^ Field perception by the cryptochromes occurs via light-driven charge separation on the cofactor of the enzyme, flavin adenine dinucleotide (FAD) followed by electron uptake by oxygen.^37,41^ Further realization of field effect in the framework of light-induced electrical reaction is apparently not related directly to cryptochrome signaling pathway, it is rather acting via elevation of reactive oxygen species (ROS). ROS can interact with calcium channels, making an extra elevation of cytosolic Ca^2+^ concentration in the light. ROS-sensitive low-selectivity channels of plasma membranes with calcium conductivity were described and characterized electrophysiologically in plants, but the genes of corresponding proteins have not been revealed yet.^42^ A similar mechanism for the formation of radical pairs as a result of absorption of light quanta is known for other flavoprotein blue light receptors – phototropins, as well as for various natural photosynthetic reaction center proteins.^43,44^ One can assume the contribution of such proteins to the perception of ELF magnetic field, however, experimental evidence is lacking to date.

Other potential targets of extremely low-frequency ELFMF capable of affecting calcium concentration could be calcium channels themselves. The sufficiency of the calcium channel structures for activation in MF has recently been shown in some works on animal cells. Particularly, such a feature has been demonstrated for TRPC1 and other uncharacterized representatives of TRP channel family covering low-selectivity ion channels capable of reacting to a great variety of environmental stimuli.^45–47^ Such channels have not been shown in higher plants yet, but recently a potential TRP channel capable of calcium conduction was discovered in green alga *Ulva compressa*.^48^

The alternative mechanism of H^+^-ATPase activity change under MF is regulation of its activity by photosynthetically synthesized sugars. Some works showed that low strength low-frequency MF is capable of affecting photosynthetic processes in plants.^5,7,9,10^ In our recent works, the effect of the field with analogous parameters was shown to be capable of increasing efficiency^12^ and progression rate^11^ of light reactions of photosynthesis in wheat seedlings. The field-induced enhancement of proton leakage across the thylakoid membrane, as well as the stimulation of an additional H^+^ flux caused by an increase in Ca^2+^ concentration due to the activation of the H^+^/Ca^2+^ antiporter, were suggested as key mechanisms.

## 5. Conclusion

On the whole, the results of the present work demonstrate the influence of the extremely low-frequency MF on light-induced electric reactions in wheat plants. This effect can be realized through the action of the extremely low-frequency MF on the signaling systems that regulate the intracellular concentration of Ca^2+^ and the activity of H^+^-ATPase. MF-induced changes in the electric potential can have a certain value for the functioning of a plant cell, since it performs a number of essential functions. The most significant ones are regulation and energization transmembrane substance transport and signal transduction. In this regard, the revealed effect of the extremely low-frequency MF on electric reactions should be taken into account when analyzing the mechanisms of the impact of MF on plants.
